# Effect of Granite Powder Grain Size and Grinding Time of the Properties of Cementitious Composites

**DOI:** 10.3390/ma15248837

**Published:** 2022-12-10

**Authors:** Agata Stempkowska, Tomasz Gawenda, Adrian Chajec, Łukasz Sadowski

**Affiliations:** 1 Department of Environmental Engineering, Faculty of Civil Engineering and Resource Management, AGH University of Science and Technology, Mickiewicza 30 Av., 30-059 Cracow, Poland; 2 Department of Materials Engineering and Construction Processes, Wrocław University of Science and Technology, Wybrzeże Wyspiańskiego 27, 50-370 Wroclaw, Poland

**Keywords:** cement paste, granite powder, milling

## Abstract

The purpose of this article is to determine the effect of granite powder grain size and grinding time on the properties of cement paste. A series of cement pastes modified by the addition of granite powder were made and the properties of the fresh mixtures and the mechanical properties of hardened pastes were studied. Based on the study, the best results, from the point of view of the application of granite powder in cementitious composites, were obtained for a sample with granite powder ground for 3 h, in which 50% of the particles were smaller than 4 μm, and 90% were below 20 μm. Compressive strength of 55 MPa and flexural strength of 6.8 MPa were obtained on this sample after aging for 28 days. To confirm the validity of using granite powder as substitute materials, additional tests such as scanning microscopy with elemental analysis (SEM, EDS) and infrared (FTIR) studies were performed.

## 1. Introduction

One trend in the development of the modern construction industry is their transition to cost-effective production technologies, while ensuring the high quality of building materials and structures. Currently, there is a significant increase in the use of industrial waste, caused by the sharp rise in energy prices and the need to expand the raw material base. This trend is noticeable worldwide [1,2]. Portland cement is most widely used in the construction industry, the most energy-intensive component of which is cement clinker, so it is desirable to conduct research on technologies that ensure a significant reduction in clinker content while maintaining adequate performance of cementitious composites. Cement is the costliest component in concrete production, both economically and energetically [3,4]. It is therefore reasonable to reduce the consumption of binders in concretes and regulate their structural and technical properties by introducing active mineral additives and fine-grained fillers of various origins. Among other things, researchers are trying to reduce the amount of cement in cement mixtures by replacing it with supplementary cementitious materials (SCMs). 

When looking for opportunities to improve the environment, it is important to test alternative material solutions (such as the use of marble, limestone or granite powders). It is observed that the addition of granite powder can reduce the cement content of mixtures. The paste substitution method has been found to be a promising way to produce high-performance eco-friendly composites, and also contributes to higher waste utilization and lower cement content [5,6,7,8,9,10]. There are literature data on the use of metakaolin, silica powder and nanosilica as cement replacements [11]. In addition, production waste from thermal power plants, metallurgical enterprises, and non-metallic material manufacturing companies are used as fillers [12,13,14]. In the literature, one encounters studies of the effects of fineness and content of various materials on cement hydration, permeability, pore structure and fractal dimension of concrete [15,16] Fillers can serve various functions, including as immobilizers of harmful and hazardous substances. 

There are new methods for advanced processing of industrial sludge using a closed-cycle drying process. These methods contribute to increasing the amount of hazardous waste used in the production of construction materials [17,18,19,20]. Most often, fine-crushed waste mineral filler actively participates in the curing and structuring of mixtures. Under conditions of the gradual depletion of natural resources and worsening environmental problems, an important direction in concrete and mortar production is the development of materials using waste materials from rock processing. Granite powder as a waste material is a promising material for use in concrete, similar to pozzolanic materials. The highly dispersible mineral component fills the space between grains and forms a rigid structure, improving the density and rheological properties of cement paste [21,22,23,24]. Stone-crushing plants collect a large amount of granite powder, which is captured by filters. In many cases, granite powder is currently not used and is deposited in landfills. As a result, the use of technogenic waste in the production of construction materials is becoming more common, and the development of energy production technologies is accelerating. Tests to use granite powder in cement mixtures have been going on for several years, and the number of studies conducted for cement mixtures with this additive is steadily increasing. Studies are being conducted relating to the partial replacement of fine aggregate in cement mortars. It has been found that the addition of granite powder (40%) improves the compressive strength of mortars, but at the same time, reduces the flexural tensile strength of mortars. It was also observed that the addition of granite powder increased the drying shrinkage and decreased the water absorption of mortars [25]. The addition of granite powder also increases the filling effect in composites [26]. Moreover, the addition of granite powder in concrete can reduce the energy consumption of vibrators and help reduce construction time. Granite powder, compared to marble powder, shows a better effect on the properties of self-compacting concrete [27]. Interesting studies were conducted with the addition of very finely ground granite-powder particles (nanometer-scale grain size). It was observed that replacing 10% of cement with nanoparticles of granite powders increased the mechanical properties of the composites [28]. 

The authors of this publication conducted a study on the effect of granite powder granulation on the mechanical parameters and microstructure of cementitious composites. The proper granite powder fineness and surface development has an impact on the reactions taking place in the cement paste and, consequently, on its parameters. A novelty is the subject of grinding granite powder to optimize the properties of the mixture and cementitious composite.

## 2. Materials and Methods

### 2.1. Granite Powders

A fine granite fraction with the chemical composition presented in Table 1 was used for the study.

**Figure 1:**
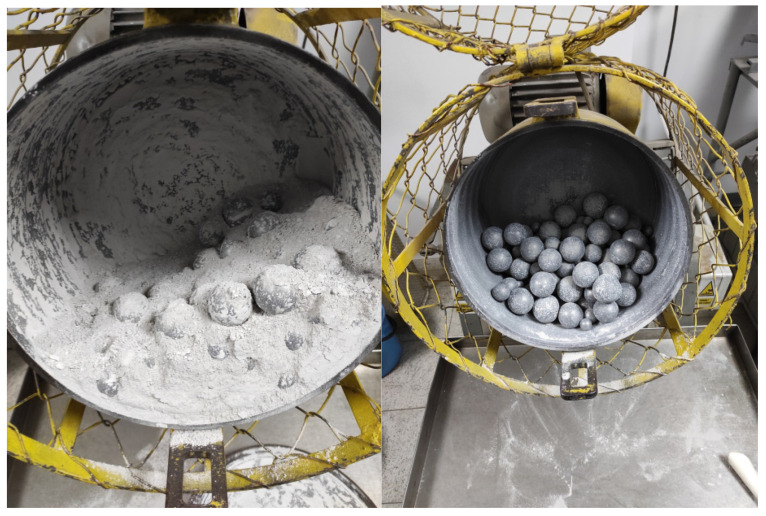
Ball mill-laboratory scale.

**Figure 2:**
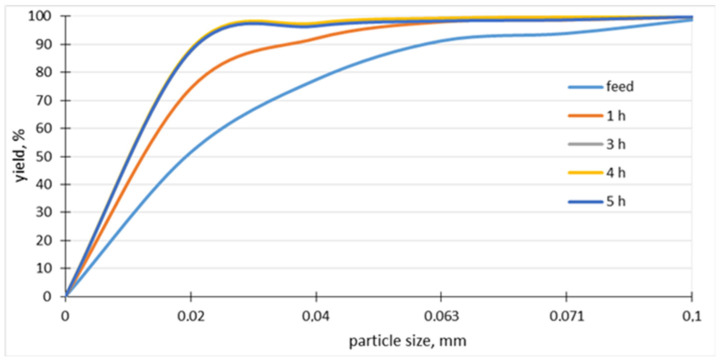
Grain composition curves of feed and grinding samples obtained with analytical sieves on wet method.

A study of the granite grinding process in a ball mill has been carried out (Figure 1). The ball mill consists of a roller with a diameter of 305 mm and a length of 200 mm, which performs 60 revolutions/min. The mill has a grinding mass of 18 kg in the form of steel balls ranging in size from 15 mm to 38 mm. The degree of filling the mill with balls is about 27%. The rotation of the mill is 78% of the critical rotation, which causes the grinders to work on the material with an abrasive force combined with impact. For grinding tests, 4 granite samples weighing 2 kg each and with a feed grain size of 0–0.2 mm were used, with 99% of the material passing through a 0.1 mm sieve and a mean grain size of d50 of about 0.02 mm The samples were ground at times of 1, 3, 4 and 5 h. Analyses of grain composition were performed using analytical sieves by the wet method and product below 0.02 mm by laser diffraction. The grain composition curves of the feed and grinding products obtained by analytical sieves are included in Figure 2.

Samples with grinding times of 1, 3 and 5 h were selected for further testing. For comparison purposes, a reference paste and those with granite powder without grinding were also made.

### 2.2. Compositions of Cementitious Paste and Preparation of Samples

To compare the effect of time of grinding of granite powder on the properties of cementitious composites modified with this powder, we prepared five series of cementitious pastes. The reference series was prepared with only 100% cement (CEM I 42.5R; Górażdże, Poland). In the other series, we replaced 20% of the amount of cement in the mix with the addition of prepared granite powder. The name GP0H describes granite powder without any grinding (natural powder), for other granite powders (GP1H, GP3H, and GP5H) we used a grinding technique to achieve more fine grains of powder—we ground GP 1, 3 or 5 h (those numbers are the names of each sample of GP). Detailed compositions of each research series are presented in Table 2.

To prepare samples of cementitious pastes modified with the addition of ground granite powder, we used the typical mixing procedure described in EN 1015-3 [29]. In the first stage, we mixed all the dry ingredients (cement and granite powder) for 90 s. After that, we added the needed amount of water (based on Table 2). Then, we mixed all ingredients (2nd stage of mixing) twice for 90 s with a 30-s break. Then, we investigated the fresh properties of cementitious pastes and after that, we cast the beams in plastic molds (40 × 40 × 160 mm). After 24 h, we removed the beams from the molds and started curing time until the time of the testing of mechanical properties of cementitious pastes procedure described in EN-1015-11 [30]. 

### 2.3. Experimental Techniques

#### 2.3.1. Particles Size Distribution of Granite Powders

Due to the limited scope of wet sieve analysis, a laser particle meter was used to measure the grain distribution of granite powders, using the Malvern Master Sizer 2000 (Malvern, UK). In the laser analyzer, the laser light beam shining through the measuring cell was diffracted onto the particles in proportion to their size. When the laser light encountered a population of grains, the volumetric distribution of their size was expressed by the intensity of the distribution of light diffracted on them. According to the principle of diffraction, small particles refract the light of the laser beam at larger angles, and larger particles are the source of smaller angles of deviation from the axis of the light stream, while the intensity of the light stream is proportional to the content (number) of individual particles. The resulting diffraction image is identified by an array of light-sensitive detectors, and the resulting signals are used to calculate the grain size distribution. 

#### 2.3.2. Determination of the Consistency of the Fresh Cementitious Composites 

To investigate the consistency of cementitious pastes we used the slump spread method. This method was conducted based on the EN 1015-3 standard [29]. We cast the cementitious pastes into the prepared measure container. After the compaction of the paste, we removed the container and shook the measuring table 12 times. After that, we measured the spread of the slump of cementitious pastes. 

#### 2.3.3. Determination of the Basic Physical Properties of the Hardened Cementitious Composites 

The basic physical properties of cement pastes, such as bulk density, porosity and water absorption, were determined. The tests were carried out in accordance with the requirements specified in the procedure PN-B-04500:1985 [30]. Determination of bulk density requires drying the samples to a constant volumetric mass (the difference between measurements over 24 h should not be greater than 1.0%), and then measuring the dimension of the sample accurately. To determine the water absorption (*W_a_*) of the samples, they were placed in water and then weighed every 24 h to determine when the samples were fully saturated (difference in sample weight <1.0%). After determining the amount of water absorbed by the samples, water absorption was calculated. Water absorption was determined from the weight of the dry sample (*m_d_*) and the weight of the wet sample (*m_w_*) according to the following formula:

(1) $$W_{a}=\frac{m_{w}-m_{d}}{m_{d}}100\;[\%]$$

Volumetric porosity (*P_v_*) was determined from the ratio of the volume of air pores (*V_p_*) in the test material to the volume (*V*) of the test sample based on the equation:

(2) $$P_{v}=\frac{V_{p}}{V}100\;[\%]$$

#### 2.3.4. Determination of the Basic Mechanical Properties of the Hardened Cementitious Composites 

To determine the basic mechanical properties of the hardened cementitious composites, destructive tests of the specimens were performed in terms of compressive strength test and flexural tensile strength test. The speed of load for compressive strength was 0.5 MPa/s, and for flexural tensile strength it was 0.05 MPa/s. Tests on beams with dimensions of 40 mm × 40 mm × 160 mm were carried out in accordance with the requirements specified in the procedure PN-EN 1015-11 [31].

#### 2.3.5. Microscale Composite Testing 

Sections of the supplied concrete plastics were taken at random and analyzed on a scanning microscope. The research was carried out using a field emission scanning electron microscope from FEI, model Nova NanoSEM 200 (Lincoln, NE, USA). This microscope allows operation under high vacuum and operation with steam as the working gas in low vacuum mode (10–200 Pa). The magnifications achievable are from 100× to 1,000,000×. The microscope allows evaluation of material surfaces, internal structure, changes and deformations. In addition, the microscope is equipped with an EDS Octan Elect X-ray energy-dispersive spectrometer from EDAX (Mahwah, NJ, USA), thanks to which it is possible to quickly analyze the chemical composition and determine the elements (quantity and quality) of selected micro-areas on the surface of the sample, including those too small for examination by other methods. The method was used for illustrative purposes and the chemical compositions referred to the measurement site from which the data were collected.

#### 2.3.6. FTIR of Hydration Products

The spectrometer used was a Bruker Vertex 70 v (Billerica, MA, USA) with an insert cell for diffuse reflectance spectroscopy. The measurement range lies between 400 and 4000 cm^−1^. The diffuse reflectance technique was utilized, in which the incident beam was allowed to be reflected off the ground sample towards an overhead mirror upon which the diffusely scattered rays were collected and measured in the detector.

## 3. Results and Discussion

### 3.1. Grain Size of Granite Powders

**Figure 3:**
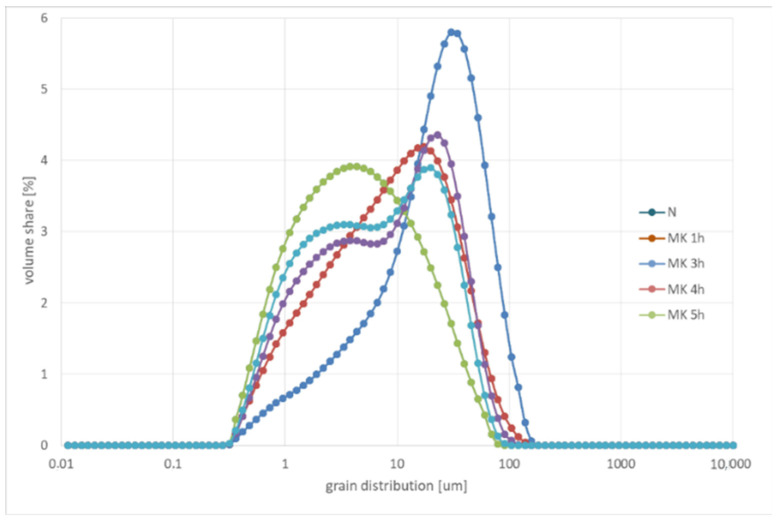
Grain distribution of samples in relation to grinding time.

Since a sieve smaller than 0.02 mm was not used for sieve analysis due to technical limitations, and the grain composition curves of grinding samples at times 3, 4 and 5 h practically overlap (Figure 2), a more accurate granulometric analysis by laser diffraction was used (Figure 3). Table 3 shows the characteristic grains of the products. Based on the analyses, it was observed that after 1 h of grinding, about 74% of the material below 20 μm was obtained. The most favorable grinding time for granite was 3 h, as 50% of the grains are smaller than 4 μm, and 90% are below 21 μm. It should be recognized that a higher grinding time than 3 h in a ball drum mill will not yield greater results in fineness, due to the formation of a paste of particles agglomerating on the grinders and drum walls by cohesion forces, and due to the occurrence of electrostatic interaction (cohesion) forces between fine particles. In the case of longer grinding time from about 4 h, abrasion of the steel grinders is observed, which manifests itself in the formation of bimodal grain distribution (Figure 3, MK 4 h and MK 5 h).

The goal of grinding granite was to achieve the most developed specific surface of the material. By reducing the particle size, we achieved greater reactivity and the ability to form cementitious phases. Table 4 shows the results of calculating the specific surface area of the samples using the Blaine method

### 3.2. Consistency

Table 5 shows the results of the Novikov cone settlement (lN) tests for cementitious composites that differ in the content of granite powder relative to the weight of cement. The study shows that the addition of granite powder in the mortar results in a lower Novikov cone settlement (lN) compared to the reference mortar (by about 10%). A similar relationship has been observed in other studies with granite powder [20,32,33].

### 3.3. Selected Physical Parameters

**Figure 4:**
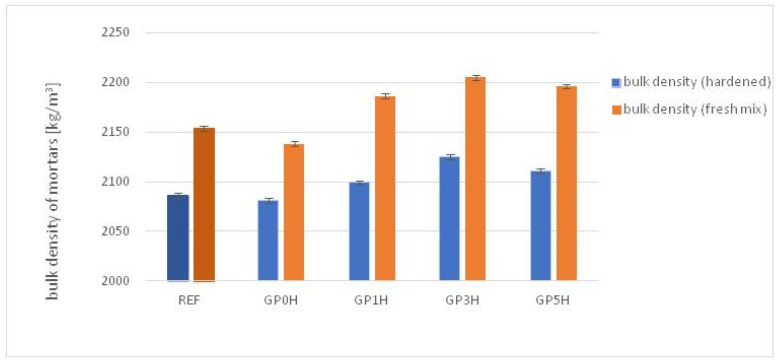
Effect of replacing the cement with the granite powder on bulk density of cementitious composites.

**Figure 5:**
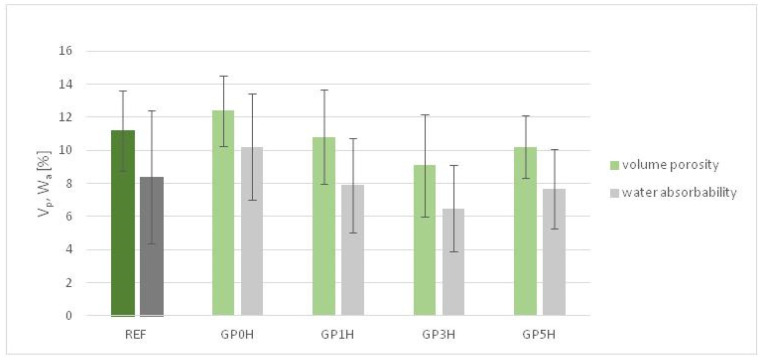
Changes in porosity and water absorption in granite powder pastes with respect to a reference sample.

Figure 4 and Figure 5 show tests of the basic physical properties of the hardened cement pastes including testing of bulk density, porosity and water absorption; the changes were compared to a reference sample. The tests are the average of six measurements taken for each composition.

The addition of granite powder increases the density of the mixture by an average of 5%, with the density increasing slightly as the grinding time of the powder increases. This is due to the development of the surface, a higher degree of fineness and the filling of small pores by micro-particles of rock. This phenomenon applies both to the fresh mixture and after hardening. Figure 5 shows the effect of replacing cement with granite powder on the volumetric porosity (*V_p_*) and water absorption (*W_a_*) of cured cement composites. Mortar with the addition of unground granite powder has a higher volumetric porosity (by about 1.5%), and water absorption (by about 2%) than the reference mortar. The values of these parameters decrease with the grinding time of granite powder; the most favorable results were obtained for a grinding time of 3 h, that is, *V_p_* of 8.5%, and *W_a_* of about 6.2%.

The differences in physical properties may be related to the fact that granite wastes differ significantly in terms of grain structure and parameters describing their surface morphology. In addition, there is a significant difference in the development of their surface (grinding time of samples) [20,31,32,33,34].

### 3.4. Mechanical Parameters: Flexural and Compressive Strengths

**Figure 6:**
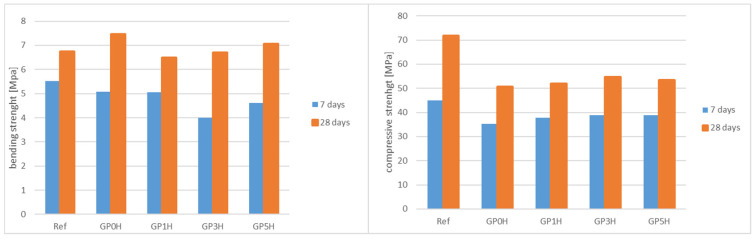
Bending and compressive mechanical strengths in hardened pastes modified with granite powder with respect to a reference sample.

Determination of the basic mechanical properties of cement pastes consisted of destructive tests—the compressive strength test and flexural strength test at the appropriate time (7 days, 28 days) after the samples were formed. Figure 6 shows the results of the determination of compressive strength and flexural strength for composites modified with the addition of granite powder. The reference sample contains 100% so its strength is higher. Since the authors wanted to show the effect of grinding on the mechanical strength of the composite, the results should be referred to sample GP0H in which 20% of the cement was replaced with unground granite powder. For samples with fine milled granite, the compressive strength gradually increases. Granite, due to its origin (crystalline plutonic rock), does not actually have pozzolanic properties. These are the ability to react silica with calcium oxide. However, by developing the surface through grinding, higher reactivity and the possibility of calcium silicates are obtained. It should be noted that this reaction is slow and can be evidenced by an increase in strength over a longer period than the standard 28 days.

### 3.5. Microscale Composite Investigation

**Figure 7:**
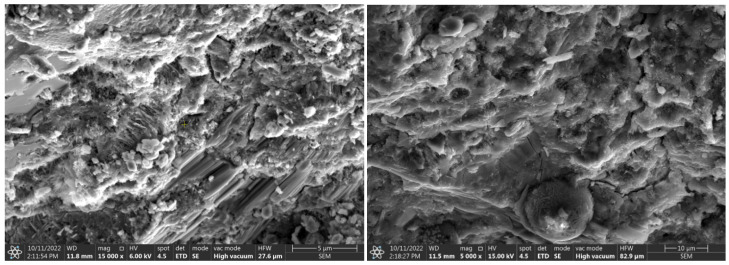
Microphotographs of the reference sample.

**Figure 8:**
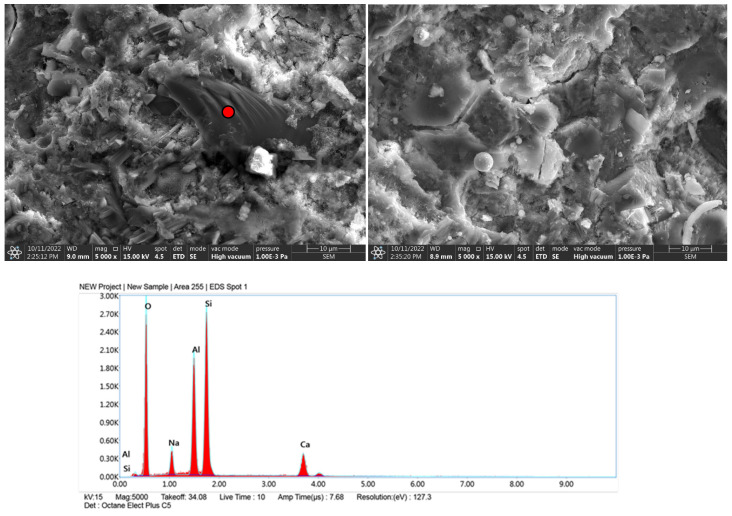
Microphotographs with EDS analysis of sample with unground granite powder.

**Figure 9:**
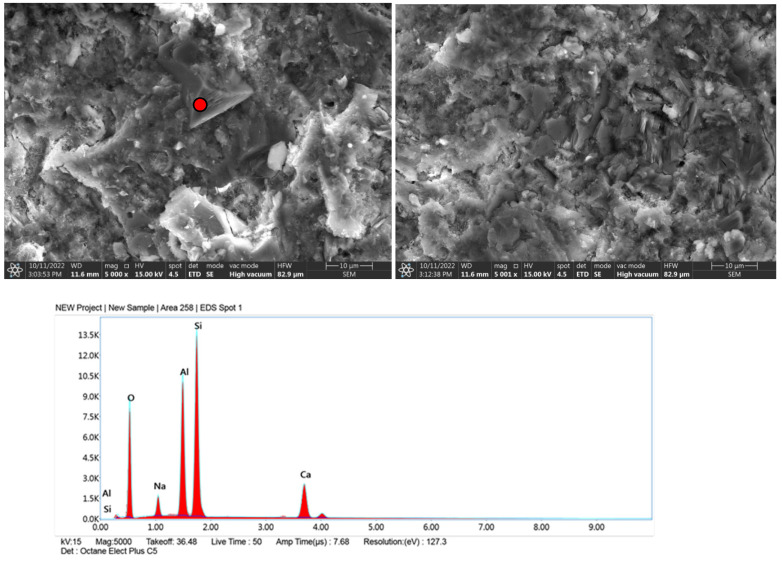
Microphotographs with EDS analysis of sample with granite powder after 3 h grinding.

**Figure 10:**
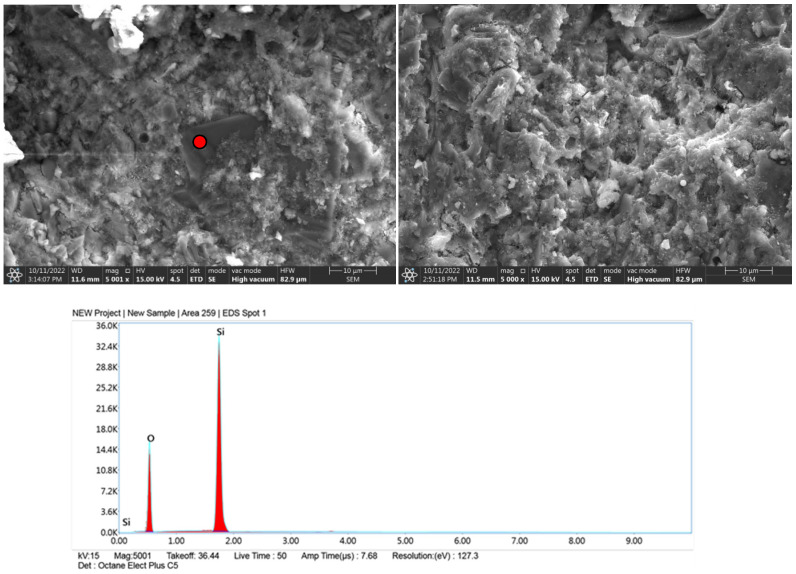
Microphotographs with EDS analysis of sample with granite powder after 5 h grinding.

Granite powder has a much smaller grain size than commonly used fine aggregates, so the partial replacement of aggregate with granite powder causes the microstructure of the cement matrix to thicken. The dominant mineral phases in the samples were C-S-H and portlandite. The former occurs mainly in a small number of needle-shaped crystals. Portlandite, which was formed by the hydration of allite (C_3_S) and belite (C_2_S), forms massive, hexagonal crystals (Figure 7-reference sample visible needle-shaped crystals of the CSH phase). In this system of cement with fine filler, granite powder does not isolate the surface of the new phases and does not block the formation of compounds during contact with the crystalline hydrates. The hydration products are deposited on the fine powder particles, and these particles form the crystallization center (Figure 8, Figure 9 and Figure 10). EDS measurements confirmed the presence of aluminates and aluminosilicates, derived from cement, and the presence of feldspar and quartz crystals in the samples. Red dots on the microphotographs indicate the location of the EDS analysis.

### 3.6. Analyses of Hydration Cementous Phases

**Figure 11:**
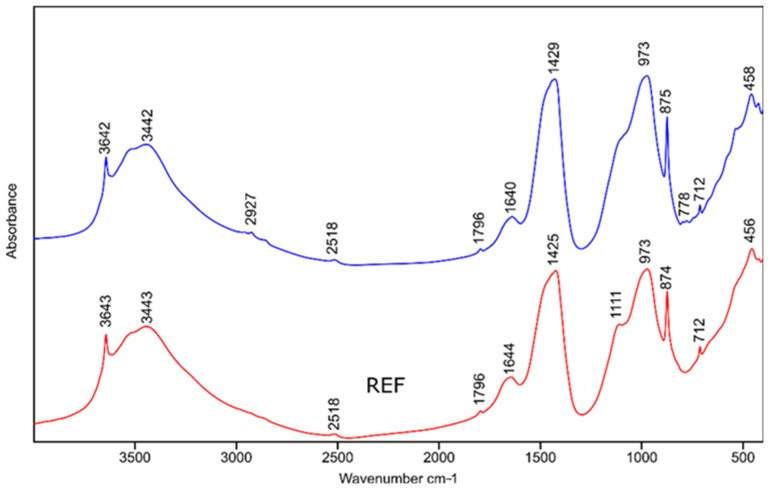
FTIR spectra showing reference sample (red) and after 3 h grinding (blue).

FT-IR spectroscopy readily highlights the presence of portlandite (Ca(OH)_2_), which is well-detected by a definite unique peak at 3640 cm^−1^. This method is also used to study the bands assigned to calcium silicate hydrate (C-S-H) as a function of curing and aging conditions [35,36]. Figure 11 and Table 6 show analyses performed in transmission mode on hydrated phases.

The main characteristic C-S-H peaks are located between 1100 and 900 cm^−1^. During aging and decalcification, these IR bands shift depending on the silica polymerization process. Assignment of the others did not show specific hydration perturbations: portlandite was detected at 3642 cm^−1^ (O-H stretching vibrations); C-S-H was characterized by main bands at 973 cm^−1^ (asymmetric Si-O stretching vibrations); for ettringite, a characteristic band was detected for O-H-1640 cm-1, and Al-O 874 cm^−1^. Peaks from S–O bonds fall at 1150-1100 cm^−1^ and overlap with Si-O vibrations for the C-S-H phase. The FTIR spectroscopy also highlighted the presence of CaCO_3_ (three distinct bands at 1425, 870 and 712 cm^−1^). The detection of CaCO_3_ was assigned to the carbonation process [37]. 

Cementitious composite systems from an application point of view can be reduced to a mixture of three components: cement; fine aggregate (powders) and water. It is a composite system in which aggregate grains are surrounded by hardened cement paste. The formation of mortar is determined by the hydration reactions of the cement. Cement is a mixture of solid phases containing primarily various types of calcium aluminates and aluminosilicates. In particular, it is: allite-C_3_S-(3CaO·SiO_2_); belite-C_2_S-(2CaO·SiO_2_); tricalcium aluminate-C_3_A-(3CaO·Al_2_O_3_); braunmillerite-C_4_AF-(4CaO·Al_2_O_3_·Fe_2_O_3_); calcium oxide (free)-CaO, anhydrite-(CaSO_4_·0.5H_2_O) [38]. The process of cement hydration is a multi-stage process. However, it seems that from the point of view of using granite powder as a cement paste component, the most important is the hydration of tricalcium aluminate. This hydration is complicated, in the presence of gypsum, ettringite is formed according to the reaction:

3CaO·Al_2_O_3_ + 3CaSO_4_·2H_2_O + 26H_2_O → 3CaO·Al_2_O_3_ ·3CaSO_4_·32H_2_O (3)

The formation of ettringite is influenced by the content of fine silica; granite is an acid rock and contains more than 65% SiO_2_ in its composition. Its fineness can significantly affect the formation of ettringite crystals [39]. The formation of ettringite whether delayed or associated with the recrystallization of this phase increases the expansion caused by the reaction of silica aggregate with alkalis, this can lead to micro-cracks of the hardened structure causing material defects [40] Granite aggregate is widely considered to be potentially reactive with cement components, so its surface should be optimally developed so that the reaction occurs as quickly as possible and reaches an equilibrium state, then material strengthening is achieved. The hardened cement paste is basically dominated by two phases: the gel phase of CSH and portlandite Ca(OH)_2_ (Figure 7, Figure 8, Figure 9 and Figure 10), in addition to which other phases formed from the hydration of tricalcium aluminate and brownmillerite are present.

A reaction desirable from the point of view of granite powder applications in grout hydration is the so-called pozzolanic reaction. This is a reaction in which a material with certain latent binding properties does not harden on its own when mixed with water; meanwhile, when finely ground, it reacts with calcium hydroxide in an aqueous environment at ambient temperature. The product of this reaction is water-insoluble hydrated calcium silicates. In general, such a reaction has the form:

SiO_2_ + Ca(OH)_2_ + H_2_O → SiO_2_∙CaO·2H_2_O (phase CSH) (4)

It should be noted that these phases contain large amounts of crystalline water in their composition, and it is water that is sometimes the main destructive factor in cementitious composites [41,42].

## 4. Conclusions 

Based on the results of cementitious composites modified with granite powder, the following conclusions were made:

- The most favorable grinding time for granite is 3 h, because 50% of the grains are smaller than 4 μm, and 90% are below 20 μm, (and below 10 μm is 75% of the material);
- The introduction of milled (optimally for 3 h) granite filler reduces the total pore volume, and reduces water absorption and penetration;
- An increase in the compressive strength of samples with the addition of finely ground granite compared to unground granite is observed;
- Images of the microstructure of cement paste with granite powder obtained by scanning microscopy indicate that the small particles act as crystallization centers, i.e., accelerate the initial stage of chemical curing.

## Figures and Tables

**Table 1:** Chemical composition of fine granite fraction.

**Chemical Composition** **%wt.**	CaO	SiO_2_	Al_2_O_3_	K_2_O	SO_3_	MgO	FeO	NaO	Fe_2_O_3_
	8.30	53.63	19.90	3.29	0.00	5.54	3.46	3.46	2.42

**Table 2:** Compositions of cementitious pastes used in research.

Series	Cement CEM I 42.5R	Water	GP0H	GP1H	GP3H	GP5H	w/c Ratio
	(Kg/m^3^)						(-)
REF	2000	800	0	0	0	0	0.4
GP0H	1600		400	0	0	0	
GP1H	1600		0	400	0	0	
GP3H	1600		0	0	400	0	
GP5H	1600		0	0	0	400	

**Table 3:** Grains’ characteristics of the samples analyzed by laser diffraction.

	Feed	Grinding Time			
		1 h	3 h	4 h	5 h
d_0.1_	2.601	1.172	0.824	1.035	0.929
d_0.5_	20.923	8.383	3.986	7.874	6.007
d_0.9_	60.233	35.036	20.757	33.771	28.984

**Table 4:** Calculated specific surface of samples.

Sample	Specific Surface (cm^2^/g)
Ref	3210
GP0H	3340
GP1H	3550
GP3H	3860
GP5H	4150

**Table 5:** Slump spread test results.

Sample	Consistency (mm)	Variability (%)
Ref	250	<5
GP0H	225	
GP1H	225	
GP3H	225	
GP5H	225	

**Table 6:** FTIR bands of the main hydrated phases.

Main Hydrated Phases	Wave Number cm^−1^	Band Detected
Portlandite Ettringite	3642	OH
C-S-H water	3443	O-H H_2_O capil
Ettringite	1640–1644	O-H
CaCO_3_	1425–1429	C-O
C-S-H	973	Si-O
Ettringite CaCO_3_	874	Al-O C-O
Ettringite CaCO_3_	712	Al-O C-O
C-S-H	456	Si-O

## Data Availability

The data presented in this article are available within the article.
